# Driveline Relocation and Vacuum-Assisted Closure for Ventricular Assist Device Driveline Infections

**DOI:** 10.3390/jcdd12060211

**Published:** 2025-06-03

**Authors:** Mehmet Cahit Saricaoglu, Melisa Kandemir, Elif M. Saricaoglu, Ali Fuat Karacuha, Ezel Kadiroglu, Mustafa Farah Abdullahi, Mustafa Bahadir Inan, Alpay Azap, Ahmet Ruchan Akar

**Affiliations:** 1 Department of Cardiovascular Surgery, Heart Center, Cebeci Hospitals, Ankara University School of Medicine, Dikimevi, Ankara 06340, Turkey; saricaoglu@ankara.edu.tr (M.C.S.); melisakandemir1999@gmail.com (M.K.); alifuatkaracuha@gmail.com (A.F.K.); elifezelkadiroglu@gmail.com (E.K.); mustaf.59@hotmail.com (M.F.A.); mbinan@ankara.edu.tr (M.B.I.); 2 Department of Infectious Disease and Clinical Microbiology, Ibni Sina Hospital, Ankara University School of Medicine, Ankara 06340, Turkey; esaricaoglu@ankara.edu.tr (E.M.S.); alpayazap@gmail.com (A.A.)

**Keywords:** left ventricular assist devices, driveline infections, microorganisms, driveline dressing

## Abstract

Background: Durable mechanical circulatory support (DMCS) infections remain a serious challenge. Ventricular assist device (VAD)-specific driveline infections (DLIs) are the most common type; however, no consensus exists on their surgical management. We aimed to define the incidence, risk factors, and microbiology of DLIs and discuss the surgical treatment modalities. Methods: We retrospectively reviewed 90 patients who underwent a left or biventricular ventricular assist device (LVAD or BiVAD) implantation with either a HeartMate 2 (Abbott), HeartWare HVAD (Medtronic), or HeartMate 3 (Abbott) in a single center between 1 March 2011 and 30 May 2023. Results: DLIs were detected in 20 (21.5%) patients during the follow-up. The mean duration of VAD support was 561.1 ± 833.2 days (1–4124 days), while it was 1277.9 ± 621.6 days in the DLI group. An extended duration of VAD support was associated with higher incidence rates of late-onset DLIs (*p* < 0.05). A younger age and lower plasma albumin levels were independent predictive factors for the risk of a DLI, with a hazard ratio of 9.77 (95%CI: 1.3–74.5) and 10.55 (95%CI: 1.40–79.35), respectively. The removal of the biofilm with velour and DL relocation through the rectus muscle combined with vacuum-assisted strategies (VAC) were performed in nine patients. One patient developed a recurrent infection, and another patient with a deep DLI subsequently received a heart transplant. No patient underwent a device exchange for an intractable DLI. Conclusions: Our results suggest that DLIs are common infectious complications after VAD implantation, which endanger patient autonomy, and impair their quality of life and overall survival. A DL relocation through the rectus muscles and VAC strategies have a role in controlling DLIs.

## 1. Introduction

The treatment approaches for advanced heart failure (AdvHF) have progressively expanded; yet, heart transplantation remains the gold-standard treatment for eligible patients with end-stage heart failure [1]. However, those who are noncandidates owing to older age, comorbid medical conditions, or contraindications—and are in need of a durable treatment—have found durable mechanical circulatory support (DMCS) devices, specifically ventricular assist devices (VADs), to be valuable alternatives that can enhance survival and quality of life in the context of AdvHF, but their utilization is frequently restricted by the adverse event profiles. Since the first introduction of DMCS, significant advances have been achieved in ventricular assist device (VAD) technology, including smaller and more reliable intrapericardial pump designs, improved hemocompatibility, and patient outcomes; however, the transcutaneous driveline (DL) continues to remain a potential port for infection, which is challenging to treat and also limits patients’ freedom [2,3,4].

A device infection remains one of the leading sources of morbidity and mortality among DMCS patients [3,5,6,7]. Driveline infections (DLIs), which typically initiate at the driveline exit site (DLES), may extend into deeper tissues, like the tunnel or pump pocket, and are classified by the ISHLT as superficial or deep infections [8]. The INTERMACS registry reported the incidence of DLIs at one year after VAD implant at 19% [9], while the long-term deep infection incidence has been estimated at 11% [10].

The recognized risk factors are metabolic disorders (e.g., diabetes, malnutrition), states of immunodeficiency, poor tunneling techniques, the exposure of the velour, and extended intensive care unit stays [4,7,11,12,13,14]. Prevention strategies, including standardized DLES care, improved DL positioning, and broad patient education, are still crucial [3,4,15,16,17,18]. The treatment strategies for DLIs include targeted hospital antimicrobial therapy in combination with surgical resection of the DLES, outpatient parenteral antimicrobial therapy, surgical debridement combined with removal of the biofilm or velour, local installation of absorbable antibiotic beads, vacuum-assisted strategies and delayed closure, closed catheter irrigation, DL relocation with an omentoplasty, muscle flaps, DL relocation to available muscles, and device exchange [3,4,5,7,10,17,19,20,21]. Antibiotics may be administered either as short-term treatment or as a lifelong chronic suppressive therapy within the framework of destination therapy. Nevertheless, the standardized treatment algorithms for DLIs are limited and generally based on expert consensus and the clinical experience of high-volume centers [7,19]. Currently, the gold-standard therapeutic solution for deep DLIs in VAD patients is heart transplantation, but the limited donor pool does not allow for this as a viable option, at least in our region. This study aimed to define the risk factors for DLIs and their microbiologic profiles and discuss surgical treatment approaches and outcomes.

## 2. Materials and Methods

### 2.1. Study Population and Protocol

**Figure 1:**
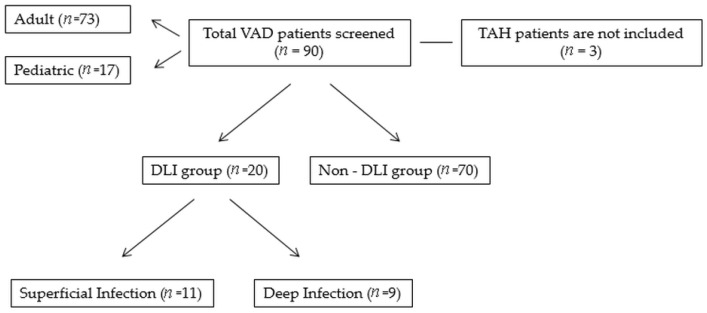
A detailed flowchart outlining the number of screened, excluded, and included patients. Abbreviations: DLI, driveline infection; TAH, total artificial heart; VAD, ventricular assist device.

We retrospectively reviewed all patients who underwent continuous-flow VAD implantation, either left ventricular assist device (LVAD) or biventricular assist device (BiVAD), at a single center. A total of 90 patients, 73 of whom were adult and 17 were pediatric patients, received VAD for AdvHF, either as a bridge to transplantation or as destination therapy, between 1 March 2011 and 30 May 2023. Three artificial heart patients were not included. Demographic and clinical information was extracted from an electronic patient health record system (Avicenna). Data were gathered about demographic information, comorbidities, VAD device type, time from VAD implantation to infection, microbiology results, number of surgical procedures, antibiotic therapy time, infectious symptoms, number of hospital readmissions, postoperative complications, reinfection rates, and overall survival. The baseline characteristics of this study’s cohort are listed in Table 1. A detailed flowchart outlining the number of screened, excluded, and included patients is presented in Figure 1.

CPB times for VAD implantation are presented as they can have implications for the etiology of infection postoperatively. Prolonged CPB times have been implicated in a systemic inflammatory response, compromised tissue perfusion, and temporary immunosuppression, all of which can heighten the risk for infectious complications. In the absence of a relevant correlation in our small study, the presentation of CPB times can assist in delineating perioperative factors resulting in infection in larger prospective studies [22].

### 2.2. Prophylactic Antibiotic Regimen and Driveline Protocol

Nasal decolonization with preoperative mupirocin ointment was applied intranasally to patients who were *S. aureus* carriers, regardless of the methicillin resistance. Standard perioperative antimicrobial prophylaxis included cefazolin and, in case of screening positive for methicillin-resistant *S. aureus* (MRSA), patients received vancomycin for prophylaxis. Antimicrobial prophylaxis was continued for a maximum of 24–48 h postoperatively.

Intraoperatively, we tunneled the DL in the sheath of the rectus muscle in the umbilical direction from the mediastinum and used a transfixing suture using 3/0 prolene at the level of the linea alba. Then, we tunneled the DL again from the rectus muscle to subcutaneous tissue to the right upper quadrant. We routinely applied strict perioperative glycemic control, avoidance of transfusion of blood products if possible, and DLES immobilization using anchoring sutures and appropriate dressings. We avoided exposing polyester velour at the exit site, and the silicone portion of the DL was always interfaced with the exit site. Due to unavailability, we could not use any anchoring device in this cohort.

### 2.3. Standard Driveline Dressing Protocol

Our patients were evaluated weekly for one month, then monthly for 1 to 3 months, then at the sixth month, and every year afterward during the follow-up period. Patients were initially taught to perform daily DL dressing changes in the early postoperative period, according to strict aseptic precautions. More frequent dressing changes, however, could be gradually reduced based on clinical status, exit-site appearance, and institutional protocol, and weekly dressing change is adequate for stable, uncomplicated cases, as supported by recent studies [23]. The DLES were to be cleansed using a chlorhexidine-based solution and subsequently covered with a sterile dressing to minimize infection risk. Silver-impregnated gauzes were used in the presence of localized skin reactions to chlorhexidine.

### 2.4. Case Definitions 

The onset of an infectious episode was defined as the time of its initial documentation in the medical record, while its resolution was marked by the completion of treatment. Recurrent infections were characterized as either new infections occurring after the cessation of therapy or as the need for therapy escalation in patients receiving long-term suppressive antibiotic treatment. Clinical signs of DLIs are presented in Table 2.

### 2.5. Imaging and Surgical Management

**Figure 2:**
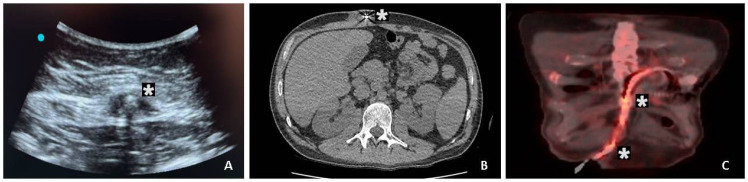
(**A**) Ultrasonography, (**B**) computed tomography, and (**C**) positron emission computed tomography images of patient who was diagnosed with driveline infection. Asterisk points out the infected region.

An initial evaluation of the extent of DLI was performed using ultrasonography in this cohort. The driveline exit site was evaluated using bedside ultrasonography using high-frequency linear transducers in all the patients to evaluate fluid collection, sinus tracts, or edema of the soft tissue. Cross-sectional imaging was used when deep or systemic infection was suspected. Computed tomography was used to examine the soft-tissue tunnel and the surrounding anatomical structures. In a few patients, especially those who had ongoing clinical features despite antimicrobial treatment, 18F-fluorodeoxyglucose positron emission tomography/computed tomography (18F-FDG PET/CT) was performed to evaluate the metabolic status and also the anatomical extent of infection. The scans were interpreted by radiologists in consultation with the infectious disease and surgical experts. Radiologists performed ultrasound-guided fluid aspiration around the DL for Gram stain and cultures. We used a 2-week course of targeted antimicrobial therapy for superficial DLES infections. Computed or positron emission tomography imaging was used in patients with suspected deep DLIs (Figure 2). If imaging showed localized abscesses associated with the DL, we performed surgical drainage, exposed the DL, and applied vacuum-assisted closure. Once the DLI was resolved, we performed DL rectus muscle relocation. Indications for driveline repositioning and muscle implantation included recurrent or persistent infection despite ≥ 6 weeks of targeted antimicrobial treatment, imaging-documented deep-tissue infection (i.e., abscess or tunnel infection), and clinical signs of systemic infection unresponsive to conservative treatment. These indications were consistently used to guide surgical decision-making in our institution.

**Figure 3:**
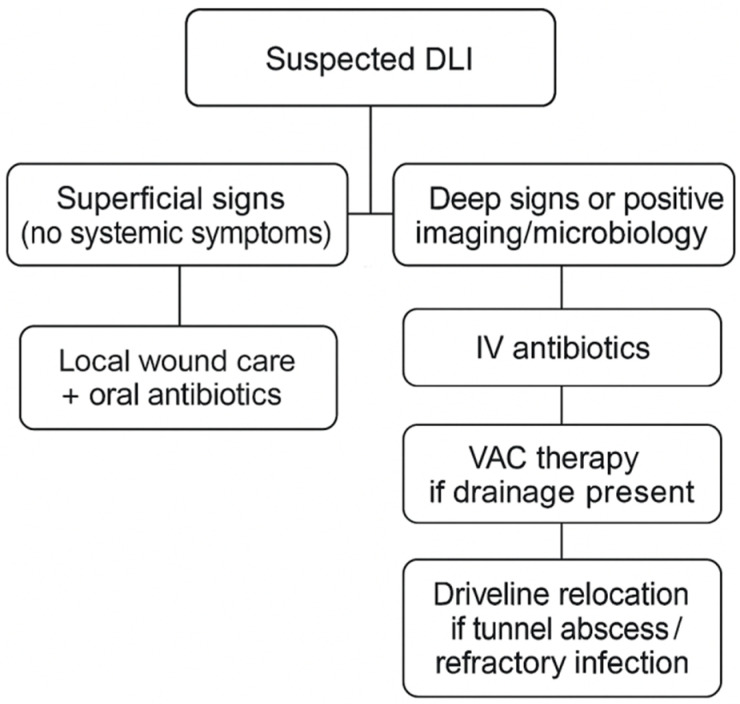
Stepwise flowchart of driveline infection (DLI) management strategy with classification, antimicrobial therapy, imaging modality, and surgical escalation. Abbreviations: DLI, driveline infection; VAC, vacuum-assisted closure.

To help delineate our stepwise approach to decision-making in infection treatment, we provide a flowchart recapitulating the diagnostic and therapeutic algorithm, as well as the selection of antibiotics, imaging procedures, surgery, and escalation criteria according to DLI severity (Figure 3). This visual aid was modified following institutional practice and recent guidelines by Seretny et al. and Trachtenberg et al. [23,24].

### 2.6. Setting

This study was approved by the Institutional Ethics Committee of Ankara University Faculty of Medicine (24 May 2024–No: 2024/286). Prior to the procedures, patients were provided with detailed information regarding the associated risks and potential therapeutic benefits. Written informed consent was obtained from all participants or their legal guardians. This study was conducted in adherence to the ethical standards set forth in the World Medical Association Declaration of Helsinki.

### 2.7. Statistical Analysis

Statistical analyses were conducted using descriptive statistics, including mean, standard deviation, median, and minimum and maximum values. Group comparisons for categorical variables were performed using a chi-square test or Fisher’s exact test, while continuous variables were analyzed using Student’s *t*-test. For non-normally distributed continuous or ordinal variables, a Mann–Whitney U test was employed. Kaplan–Meier survival analysis and a log-rank test were applied for univariate analysis, whereas multivariate analysis was conducted using Cox proportional hazards regression. Variables with a *p*-value < 0.25 in the univariate Cox regression, along with known independent risk factors for driveline infections, were included as candidates in the multivariate model. The final model incorporated variables with a *p*-value < 0.05, which was deemed statistically significant. Receiver operating characteristic (ROC) curves were utilized to evaluate the diagnostic performance of the methods. An area under the curve (AUC) of 0.50 indicated no discriminatory ability. Youden’s index was applied to identify the optimal cut-off values for the diagnostic methods. The AUC and corresponding 95% confidence intervals (CIs) for each variable were calculated and statistically compared. Statistical analyses were performed using SPSS version 29.0 (IBM SPSS Statistics, Chicago, IL, USA).

## 3. Results

During the study period, a total of 90 patients underwent a VAD implantation, with 39 receiving a HeartWare HVAD (Medtronic, MN, USA), 38 receiving a HeartMate 3 (HM3, Abbott, North Chicago, IL, USA), 10 receiving a HeartMate 2 (HM2, Abbott, North Chicago, IL, USA), and 3 receiving an HVAD or HM3 as a BiVAD configuration.

During the follow-up with the VAD patients, DLIs were detected in 20 (21.5%) patients. Additionally, a total of 12 patients (13.3%) also received a concomitant cardiac implantable electronic device (CIED); however, no cases of cardiac device-related infective endocarditis (CDRIE) were identified during the follow-up. The mean age of all the patients was 43.6 ± 17.7, and the mean age was 31.5 ± 15.9 in the DLI group (*p* < 0.05). In 15 (75%) patients in the DLI group, dilated cardiomyopathy was the etiology for their AdvHF. However, in the non-DLI group, 27 (38.7%) patients had dilated cardiomyopathy, 39 (57.14%) patients had ischemic cardiomyopathy, and 3 (4.2%) patients had other reasons for the etiology of their AdvHF (*p* < 0.05). Only 2 (10%) patients had hyperlipidemia in the DLI group, and 40 (57.1%) patients had hyperlipidemia in the non-DLI group (*p* < 0.05). Among the adult patients, ischemic and dilated cardiomyopathies were the most common underlying diagnoses. In contrast, the pediatric patients more frequently presented with congenital heart diseases or specific forms of cardiomyopathy, consistent with the previous literature [25].

The mean plasma albumin level was detected at 28.8 ± 2.4 g/dL in the DLI group and 33.6 ± 5.3 in the non-DLI group (*p* < 0.05). For the ROC analysis, the cut-off values were 52 years for age and 30.4 g/dL for the plasma albumin level. In the Cox proportional hazards model, a younger age and plasma albumin levels were independent predictive factors for the risk of a DLI, with a hazard ratio of 9.77 (95%CI: 1.3–74.5) and 10.55 (95%CI: 1.40–79.35), respectively (Table 3).

**Figure 4:**
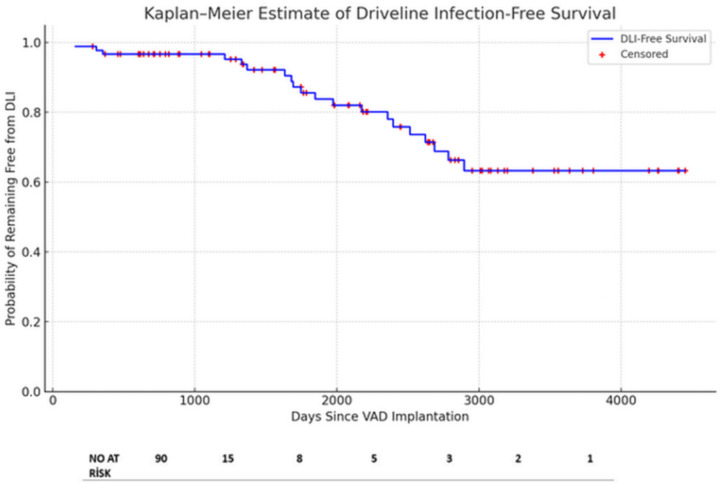
Kaplan–Meier estimates of driveline infection-free survival for all VAD patients (*n* = 90), with censoring where indicated for infection-free patients during follow-up. Abbreviations: DLI, driveline infection; VAD, ventricular assist device.

Our mean duration of the VAD time was 561.1 ± 833.2 days in all patient groups. On the other hand, the mean duration of the VAD time was 1277.9 ± 621.6 days in the DLI group. This shows a statistically significant relation between the risk of a DLI infection and the total duration of the VAD time (*p* < 0.05). This association was statistically significant (*p* < 0.05). The median time from the VAD implantation to the first DLI admission was 513 days (IQR = 404). The figure for DLI-free days is presented in Figure 4. This Kaplan–Meier plot includes all the patients and depicts right-censoring for the patients who had not developed a DLI during the follow-up.

### 3.1. Culture Results

**Figure 5:**
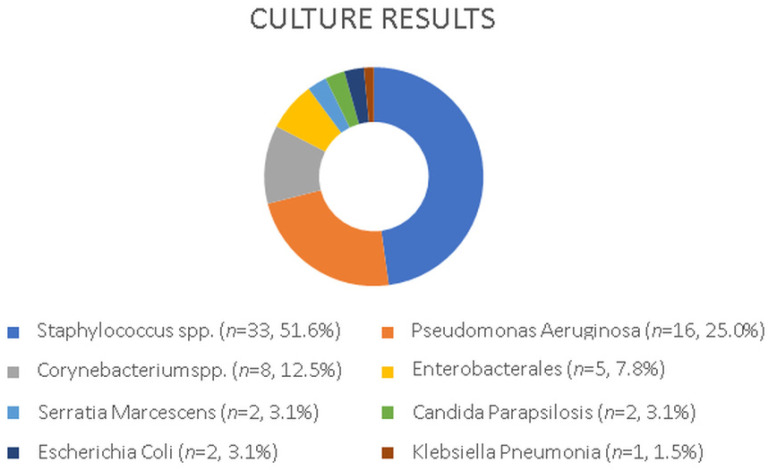
Microorganisms isolated from swab cultures, showing absolute counts and corresponding percentages.

Sixty-four isolates were identified from 55 drainage cultures of the 20 patients clinically diagnosed with a DLI. The most common isolated microorganisms were *Staphylococcus* spp. at 33 (51.6%), and Pseudomonas aeruginosa at 16 (25.0%). Among the staphylococcal isolates, *Staphylococcus aureus* accounted for 23 (69.7%) of them, while coagulase-negative *staphylococci* (CoNS) were present in 10 (30.3%). The prevalence of methicillin resistance was observed to be 36% (14/23) in *S. aureus* and 20% (2/10) in CoNS. *Pseudomonas aeruginosa*, identified as the second most frequently detected microorganism, was isolated from only from three patients’ cultures. However, *Pseudomonas* spp. accounted for recurrent and persistent DLIs. Other detected pathogens were *Corynebacterium* spp. (8, 12.5%); and *Enterobacterales* (5, 7.8%), including *Serratia marcescens*, *Escherichia coli* and *Klebisella* spp. *Candida parapsilosis* (2, 3.1%) was isolated in two cases following recurrent DLIs. A bloodstream infection was observed in only two patients as a complication of a DLI. The identified microorganisms from the DL cultures are presented in Figure 5.

### 3.2. Outcomes and Infection Management

**Figure 6:**
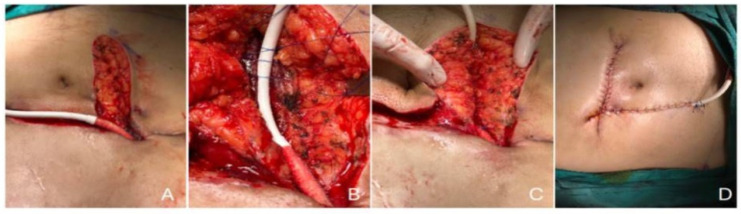
(**A**) Debridement of the driveline tunnel and preparation of the new tract; (**B**–**D**) repositioning and embedding of the driveline inside the rectus muscle fibers beneath the fascia.

Driveline infections were managed in collaboration with the DMCS team, which included infectious disease specialists. According to the swab culture results, 20 patients received targeted antibiotic therapy. The clinical response guided the duration of antimicrobial treatment, along with the type of infection (superficial or deep), pathogen(s), and the opinion of an infectious disease expert. The duration of antimicrobial treatment was meticulously determined, with at least two weeks for superficial DLIs and at least six weeks for deep DLIs. In addition to antibiotic treatment, surgical debridement was performed in 10 patients. Nine of them had a DL relocation followed with a vacuum-assisted closure (VAC) until their culture results were negative. Additionally, muscle relocation was defined as the repositioning and embedding of the driveline inside the rectus muscle fibers beneath the rectus fascia. The surgical team cleansed the DLES with chlorhexidine and hypochlorous acid and performed dressing changes according to the condition of the exit site, typically daily in the early postoperative period and less frequently thereafter, ensuring the highest standards of aseptic care (Figure 6). All the patients responded to the DL relocation and VAC therapy except one patient, who had a resistant DLI despite the recurrent surgical and antibiotic therapies. This one patient received a heart transplant.

Among the 20 driveline infection patients (Table 2), 12 (60%) retrospectively fulfilled the 2024 ISHLT consensus criteria for a complicated DLI based on the findings of a deep-tissue infection that necessitated surgical intervention and/or systemic infection, such as a bloodstream infection.

## 4. Discussion

DMCS has been proven to be a highly effective treatment for AdvHF patients [2]. As the number of cases and the duration of VAD support increase, a new spectrum of long-term complications, including late infections, has emerged. This finding is in accordance with more recent studies carried out by Vadalà et al., which showed that despite progress in VAD technology and patient management, complications like major bleeding and infection remain prevalent and significantly impact the long-term outcomes in patients receiving VADs [26]. A DLI is the most frequent infectious complication during long-term follow-up, with a prevalence of 14–28% [9,10,27,28,29]. In this study, we examined the incidence of DLIs in a single institutional cohort of 90 patients with VADs.

The DLI incidence rate observed in our analysis, which was 21.5%, is in line with previous studies [9,10,27,28,29]. Gordon et al. [11] reported that the risk of VAD infection peaked at 18 days post-surgery, and was lower and constant after 60 days. Goldstein et al. [9] showed that the peak incidence of percutaneous site infections occurred at 6 months, and Spano et al. [27] reported the peak incidence of a VAD-specific/related infection was at 4 months postoperatively. In contrast to the discrepancies observed among observational studies regarding the peak DLI incidence, our data indicate that the peak DLI incidence occurred approximately 18 months after a VAD implantation. The median number of days from an LVAD implantation to the first DLI was 513 (IQR = 404). We found a clear relationship between the risk of DLIs and the total duration of the VAD time (*p* < 0.05). In this context, an extended duration of VAD support was associated with higher incidence rates of late-onset DLIs. This introduces the possibility of reverse causality, whereby the longer duration of support itself is a causative factor in the development of DLIs. Those with more prolonged VAD exposure are, by definition, at an increased cumulative risk of infection due to the greater transcutaneous driveline time, biofilm exposure, and device–tissue interface stress. Although a younger age and hypoalbuminemia were identified as independent predictors of a DLI in our multivariate model, it is possible that these risk factors overlap with the support time, rather than existing as independent predictors. Yet, the fairly broad confidence intervals presented for these predictors (e.g., HR for albumin: 10.55; 95% CI: 1.40–79.35) raise the possibility of the overfitting of the model due to the limited number of events. This statistical imprecision restricts the generalizability of our results. The findings must thus be interpreted with caution and validated in larger populations or multicenter trials. These results are in agreement with the earlier literature that described a greater infection burden in patients with longer VAD support durations [23].

Advanced age does not appear to be a risk factor for VAD-specific complications. However, Goldstein et al. found that younger age was a significant risk factor for DLIs [9]. Similarly, we also identified a significant relation between younger age and the risk of DLIs (*p* < 0.05). An age under 52 years was an independent risk factor for DLIs [H.R.: 9.77 (1.28–74.51)] in our study population. This finding is thought to be due to the higher activity rates and, therefore, increased risk of DLES microtrauma in a younger population because of shearing traction or torsion injury.

Raymond et al. [30] reported a strong correlation between a higher body mass index (BMI) and continued weight gain throughout the course of LVAD therapy and the risk of DLIs. However, we could not detect a similar correlation in our cohort (*p* > 0.05). Furthermore, based on the DLI and non-DLI group comparison, the presence of diabetes, hypertension, or renal dysfunction was not found to serve as a risk factor for the development of a DLI after VAD support. However, we found that a plasma albumin level under 30.4 g/dL was a device-independent risk factor for a DLI. This may indicate the essential role of adequate plasma albumin levels for better wound healing, as they support tissue repair and immune function.

Previous microbiological studies have revealed the predominance of *Staphylococcus* and *Pseudomonas aeruginosa* in patients with DLIs during DMCS [10,11,27,29,31]. Biofilm dissemination and migration along the DL are critical in deep DLIs. As demonstrated previously, *Staphylococcus* spp. is recognized as a biofilm producer and accounts for a higher percentage of the initial pathogens in DLESs [11,32], which correlates with our findings. *Pseudomonas aeruginosa* becomes more common and complex to treat over time, accounting for mostly progressive, deep DLIs and pump pocket infections. Although *P. aeruginosa* was identified in the cultures of three patients, it was isolated in 25% of swab cultures despite all the treatment modalities. The failure to achieve a sufficient microbiological and clinical response in *P. aeruginosa* infections, the second most frequently detected pathogen in this study, due to its biofilm formation capacity despite treatment, is quite concerning. In cases where antibiotic treatment of DLIs was ineffective or inadequate, surgical debridement or the transposition of the driveline was achieved. A simple incision and drainage with circumferential tissue and biofilm removal around the DL were performed in one of ten patients. However, the remaining nine required rectus muscle relocation in situ or with the transposition of the driveline ipsilateral or contralateral site. Following the removal of the coating with biofilm—with velour coating for HVAD patients—the placement of the driveline into the sheath of the rectus muscle was established. This was followed with vacuum-assisted closure (VAC) until the culture results were negative. One of these nine patients was readmitted due to recurrent infection after a 1-year follow-up (11.1%). As stated, similar studies of rectus muscle relocation with or without wound VAC therapy may provide better outcomes. Juraszec et al. [21] also reported promising results that only 20% of patients treated surgically developed reinfection during follow-up.

The early clinical diagnosis of a DLI is supported by laboratory data, microbiology, and imaging findings, on which the stage-related management of a DLI depends. According to the current ESC guidelines and EHRA consensus surveys, multimodal imaging has now become central to the diagnosis of infective endocarditis (IE), especially in suspected device infection. Transthoracic echocardiography (TTE) and transesophageal echocardiography (TEE) remain the first-line diagnostic techniques for the detection of vegetations, abscesses, and perilead lesions. Cross-sectional and nuclear imaging modalities can yield valuable incremental prognostic information. Computed tomography (CT) helps to define structural complications, while 18F-FDG PET/CT improves the sensitivity of the detection of prosthetic valve and device infections because of the detection of early metabolic changes. Furthermore, a 99mTc-HMPAO-labeled white blood cell (WBC) SPECT/CT was recently validated as a specific imaging technique for IE diagnosis and is now incorporated into ESC diagnostic algorithms. Holcman et al. showed that this approach had a high specificity in difficult cases of infective endocarditis (IE), and Traykov et al. showed its growing clinical application in different European centers [33,34]. The integration of these modalities into standard diagnostic algorithms allows for early intervention and improves patient stratification. In addition, ultrasound may help detect the presence of abscess or localized purulent collection along DLs. Computer tomography (CT) is the reference imaging method for staging DLIs. Positron emission tomography (18F-FDG PET-CT) allows for the precise localization of infection and the assessment of the extent of a DLI [4,35].

A prior meta-analysis conducted by Bauer et al. [19] demonstrated that by 12 months post-VAD implantation, device exchange offered no significant benefit for reducing the overall mortality or infection recurrence compared to non-exchange approaches. In this study, we tried most of the non-exchange modalities, including targeted antibiotics, recurrent debridement procedures, the removal of the velour and biofilm, DL relocation through the rectus muscle, and VAC strategies where needed. Repeated counseling of patients and caregivers was ensured after combined surgical management.

An increasing number of patients with chronic heart failure and long-term VAD support also have cardiac implantable electronic devices (CIEDs), including implantable cardioverter–defibrillators (ICDs) and cardiac resynchronization therapy defibrillators (CRT-D). They carry a high risk of localized and systemic infections, such as lead-dependent infective endocarditis (LDIE), and are difficult to manage [22]. Ząbek et al. recently showed that while a transvenous lead extraction (TLE) in LVAD carriers can be accomplished successfully, the mortality still remains high in patients with infectious indications despite complete procedural success. On the other hand, a TLE for non-infectious indications seems to be safer, albeit with limited data [22]. In the PCHF-VAD registry, active defibrillator-containing CIEDs were found to be associated with better survival when on VAD support, particularly in patients with a history of ventricular arrhythmias [36]. However, other studies have reported discordant survival benefits depending on the baseline characteristics and the timing of the CIED implantation [25]. So, while CIEDs may have a protective effect against arrhythmic death, their use in VAD patients should be individualized and, in the setting of infection, a TLE should be considered based on the current guidelines.

### Limitations

The presented study has limitations. Firstly, our study is limited by its small sample size, and the research is a single-center retrospective analysis. This caused statistical limitations when calculating the associations, such as the relation between the DLI state and comorbidities, BMI, and device types. This study also has an over-representation of men in our cohort, as do most of the cohorts treated by VAD. A further limitation of our study includes the lack of utilization of a standardized scoring system, such as the Decline Score or Modified Memorial Sloan Kettering Sharp Score, for evaluating the driveline exit site status. Although its determination was conducted based on clinical judgment and institutional standard practices, the utilization of such scoring systems would increase reproducibility and must be taken into consideration in upcoming studies. However, these limitations provide valuable insights for conducting future research in this field.

## 5. Conclusions

DLIs are common infectious complications after a VAD implantation. Currently, the prevention and control of DLIs are essential for the management of VAD patients. The surgical resection of the DLES, the removal of the biofilm and velour, vacuum-assisted strategies, rectus muscle relocation, or omentoplasty have emerged as essential adjuncts for treating DLIs, in addition to targeted antibiotics. However, no comprehensive guidelines exist for diagnosing and surgically treating DLIs. We believe this cohort will pave the way for future randomized trials, offering hope for improved therapy for DLIs; however, future technological evolutionary solutions may eliminate all drivelines and their associated complications.

## Figures and Tables

**Table 1:** Demographic and baseline clinical characteristics stratified by DLI outcome.

	All Patients (*n* = 90)	DLIs Group (*n* = 20)	Non-DLIs Group (*n* = 70)	*p*-Values
Sex—No. (%) Male	74 (82.2%)	17 (85%)	57 (81.4%)	0.502
Age—yr Mean Range	43.6 ± 17.7 10–70	31.5 ± 15.9 12–67	47.1 ± 16.7 10–70	0.01
Age group—No. (%) Pediatric Adult	17 (18.9%) 73 (81.1%)	8 (40.0%) 12 (60%)	9 (12.9%) 61 (87.1%)	0.21
BMI Mean Range	25.5 ± 5.6 13.0–40.2	24.9 ± 5.7 14.4–32.5	25.6 ± 5.6 13.0–40.2	0.77
Hypertension—No. (%)	21 (23.3%)	5 (25%)	16 (22.9%)	0.47
Diabetes—No. (%)	18 (20%)	4 (20%)	14 (20%)	0.58
Chronic Kidney Failure—No. (%)	6 (6.7%)	0 (0%)	6 (8.6%)	0.21
Hyperlipidemia—No. (%)	42 (46.7%)	2 (10%)	40 (57.1%)	0.04
COPD—No. (%)	5 (6.7%)	1 (5%)	4 (5.7%)	0.37
Previous Stroke—No. (%)	3 (3.3%)	0 (0%)	3 (4.3%)	0.99
History of Peripheral Vascular Disease—No. (%)	4 (4.4%)	1 (5%)	3 (4.3%)	0.97
Smoking History	35 (38.9%)	6 (30%)	29 (41.4%)	0.39
Previous Implantable Defibrillator—No. (%)	17 (18.9%)	4 (20%)	13 (18.6%)	0.36
Hemoglobin (g/dL), mean ± SD	10.2 ± 0.7	10.4 ± 0.2	9.9 ± 0.8	0.78
Platelet Count (×10^9^/L)—mean ± SD	112.7 ± 17.8	115.8 ± 21.2	121.0 ± 19.4	0.99
Albumin Level (g/L)—mean ± SD	32.2 ± 4.7	28.8 ± 2.4	33.6 ± 5.3	0.03
Multi-Organ Failure—No. (%)	12 (13.33%)	2 (10%)	10 (14.3%)	0.46
VAD Duration Time (days) Mean Range	561.7 ± 833.2 1–4112	1277.85 ± 621.6 149–2779	356.3 ± 773.0 1- 4112	0.001
Heart Failure Etiology—No. (%) DCMP ICMP Other	42 (46.7%) 44 (48.9%) 4 (4.4%)	15 (75%) 4 (20%) 1 (5%)	27 (38.6%) 40 (57.1%) 3 (4.3%)	0.02
LVEF% Mean Range	18.62 ± 6.30 10–35	22.11 ± 8.2 10–35	17.6 ± 5.21 10–31	0.22
Type of Device—No. (%) HeartWare HVAD HeartMate 2 HeartMate 3 BiVAD	39 (43.3%) 10 (11.1%) 38 (42.2%) 3 (3.3%)	11 (55%) 1 (5%) 8 (40%) 0 (0%)	28 (40%) 9 (12.9%) 30 (42.9%) 3 (4.3%)	0.34
CPB Times—minutes Mean Range	202.4 ± 71.9 111–433	175 ± 50.8 144–283	209.5 101–433	0.10
Intention of VAD—No. (%) Bridge to transplant Bridge to destination	77 (85.6%) 13 (14.4%)	19 (95%) 1 (5%)	58 (85.9%) 12 (11.1%)	0.16

Plus–minus values are means ± SD. The body mass index is the weight in kilograms divided by the square of the height in meters. Abbreviations: BMI, body mass index; BiVAD, biventricular assist device; CPB, cardiopulmonary bypass; COPD, chronic obstructive pulmonary disease; DCMP, dilated cardiomyopathy; DLI, driveline infection; ICMP, ischemic cardiomyopathy; LVEF, left ventricular ejection fraction; VAD, ventricular assist device.

**Table 2:** Patients’ symptoms and clinical signs in the driveline infection (DLI) group.

Number of Days from VAD Implantation to First DLI	All Patients (*n* = 20)
Median Interquartile Range	513 404
DL Infection Symptoms—No. (%) Drainage from the DLES Fever Erythema Pain in the DL	20 (100%) 5 (25%) 7 (35%) 4 (20%)
Increased Acute-Phase Reactants—No. (%)	20 (100%)
Signs of Infection by Ultrasonography—No. (%)	12 (60%)

Abbreviations: DL, driveline; DLES, driveline exit site; DLI, driveline infection; VAD, ventricular assist device.

**Table 3:** Predictive factors for driveline infection using Cox proportional hazards model.

	Univariate Model		Multivariate Model	
	HR (95%CI)	*p*-Value	HR (95%CI)	*p*-Value
Age <52 y	11.78 (1.6–88.6)	0.02	9.77 (1.3–74.5)	0.03
Hyperlipidemia Yes/No	0.14 (0.03–0.059)	0.01		
Albumin Level (g/dL) <30.35 g/dL	12.79 (1.70–95.73)	0.01	10.55 (1.40–79.35)	0.02
Etiology DCMP vs. ICMP Others vs. ICMP	3.19 (1.04–9.69) 3.47 (0.376–32.02)	0.04 0.27		

Abbreviations: DCMP, dilated cardiomyopathy; ICMP, ischemic cardiomyopathy.

## Data Availability

Data is contained within the article.

## Abbreviations

AdvHF	Advanced heart failure
BiVAD	Biventricular assist device
BMI	Body mass index
CoNS	Coagulase-negative staphylococci
DL	Driveline
DLES	Driveline exit site
DLIs	Driveline infections
DMCS	Durable mechanical circulatory support
HM2	HeartMate 2
HM3	HeartMate 3
HVAD	HeartWare
ISHLT	International Society for Heart and Lung Transplantation
LV	Left ventricle
LVAD	Left ventricular assist device
MRSA	Methicillin-resistant *Staphylococcus aureus*
MSSA	Methicillin-sensitive *Staphylococcus aureus*
PAP	Pulmonary artery pressure
VAC	Vacuum-assisted closure
VAD	Ventricular assist device
